# Supplementary material for: Motor learning induces myelin-related white matter changes revealed by MRI-based in vivo histology
Source: Commun Biol. 2026 Feb 15;9:380. doi: 10.1038/s42003-026-09712-w (PMC12992915; doi:10.1038/s42003-026-09712-w)
Supplement: Supplementary file 2 — Supplementary Information [file 42003_2026_9712_MOESM2_ESM.pdf]

Motor learning induces myelin-related white matter changes revealed by MRI-based in vivo histology

Supplementary Material

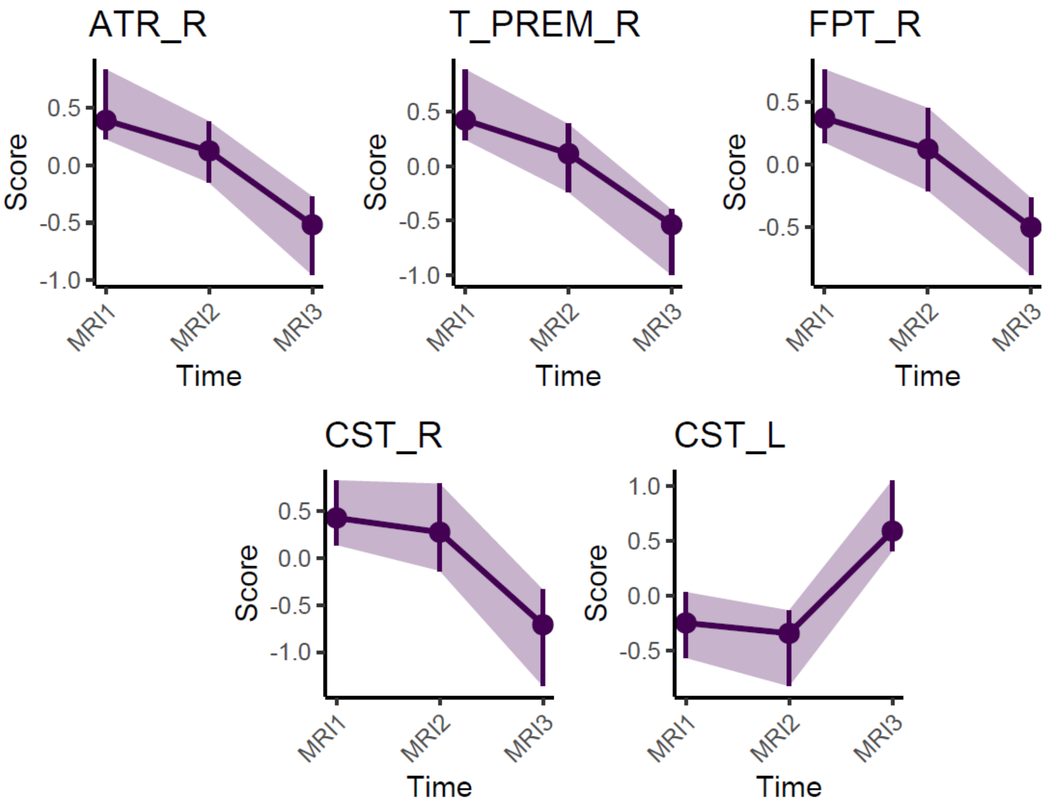

Supplementary Figure 1. Microstructural plasticity in response to motor learning in male adults (*n* = 21) across five white matter fiber tracts. Point estimates represent scores on the first principal component (PC1), with error bars indicating 95% confidence intervals (see Fig. 3, main manuscript). Female participants (*n* = 3) were excluded in this sensitivity analysis.

Abbreviations: ATR, anterior thalamic radiation; T_PREM, thalamopremotor tract; FPT, frontopontine tract, CST, corticospinal tract; LH, left hemisphere; RH, right hemisphere
