# Supplementary material for: Motor learning induces myelin-related white matter changes revealed by MRI-based in vivo histology
Source: Commun Biol. 2026 Feb 15;9:380. doi: 10.1038/s42003-026-09712-w (PMC12992915; doi:10.1038/s42003-026-09712-w)
Supplement: Supplementary file 3 — Description of Additional Supplementary Files [file 42003_2026_9712_MOESM3_ESM.pdf]

Description of Additional Supplementary File

File name: Supplementary Data
Description: Numerical source data underlying all figures and main conclusions are provided in Supplementary Data
