# Supplementary material for: Motor learning induces myelin-related white matter changes revealed by MRI-based in vivo histology
Source: Commun Biol. 2026 Feb 15;9:380. doi: 10.1038/s42003-026-09712-w (PMC12992915; doi:10.1038/s42003-026-09712-w)
Supplement: Supplementary file 5 — Reporting Summary [file 42003_2026_9712_MOESM5_ESM.pdf]

nature portfolio

Corresponding author(s): Nico Lehmann

Last updated by author(s): Aug 25, 2025

Reporting Summary

Nature Portfolio wishes to improve the reproducibility of the work that we publish. This form provides structure for consistency and transparency in reporting. For further information on Nature Portfolio policies, see our Editorial Policies and the Editorial Policy Checklist.

Statistics

For all statistical analyses, confirm that the following items are present in the figure legend, table legend, main text, or Methods section.

n/a	Confirmed	
☐	☒	The exact sample size (n) for each experimental group/condition, given as a discrete number and unit of measurement
☐	☒	A statement on whether measurements were taken from distinct samples or whether the same sample was measured repeatedly
☐	☒	The statistical test(s) used AND whether they are one- or two-sided<br>*Only common tests should be described solely by name; describe more complex techniques in the Methods section.*
☐	☒	A description of all covariates tested
☐	☒	A description of any assumptions or corrections, such as tests of normality and adjustment for multiple comparisons
☐	☒	A full description of the statistical parameters including central tendency (e.g. means) or other basic estimates (e.g. regression coefficient) AND variation (e.g. standard deviation) or associated estimates of uncertainty (e.g. confidence intervals)
☐	☒	For null hypothesis testing, the test statistic (e.g. F, t, r) with confidence intervals, effect sizes, degrees of freedom and P value noted<br>*Give P values as exact values whenever suitable.*
☒	☐	For Bayesian analysis, information on the choice of priors and Markov chain Monte Carlo settings
☐	☒	For hierarchical and complex designs, identification of the appropriate level for tests and full reporting of outcomes
☐	☒	Estimates of effect sizes (e.g. Cohen's d, Pearson's r), indicating how they were calculated

Our web collection on statistics for biologists contains articles on many of the points above.

Software and code

Policy information about availability of computer code

Data collection	No software was used for data collection.
Data analysis	FSL (topup, eddy), MRtrix3, TractSeg v2.8, NODDI Matlab Toolbox v1.0.1, MATLAB R2022b with hMRI toolbox v0.2.0 in SPM12, R v4.2.2 (lme4, ALASCA, psych, robustbase, metacor). Custom code available upon request.

For manuscripts utilizing custom algorithms or software that are central to the research but not yet described in published literature, software must be made available to editors and reviewers. We strongly encourage code deposition in a community repository (e.g. GitHub). See the Nature Portfolio guidelines for submitting code & software for further information.

Data

Policy information about availability of data

All manuscripts must include a data availability statement. This statement should provide the following information, where applicable:

- Accession codes, unique identifiers, or web links for publicly available datasets
- A description of any restrictions on data availability
- For clinical datasets or third party data, please ensure that the statement adheres to our policy

The data that support the findings of this study are available from the corresponding author upon reasonable request.

Research involving human participants, their data, or biological material

Policy information about studies with human participants or human data. See also policy information about sex, gender (identity/presentation), and sexual orientation and race, ethnicity and racism.

Reporting on sex and gender	Sex/ gender were defined based on self-reports. Both male and female participants were included (21 men, 3 women; age 19–29 years). Sex/gender was not analyzed as a covariate, as the study was not designed or powered to test sex-specific effects and no hypotheses regarding sex/gender differences were formulated. Future research with larger, balanced samples will be required to investigate potential sex/gender effects on learning-induced white matter plasticity.
Reporting on race, ethnicity, or other socially relevant groupings	Participants were recruited locally from the university community in Magdeburg, Germany. No information on race or ethnicity was collected, and analyses were not stratified by these variables. The study was not designed or powered to test for effects of race, ethnicity, socioeconomic status, or other socially relevant groupings.
Population characteristics	The study sample comprised 24 healthy, right-handed adults (3 women, 21 men; mean age = 22.2 years, SD = 3.1, range = 19–29 years; BMI mean = 23.6, SD = 2.5, range = 18.99–29.40). All participants had no history of neurological, psychiatric, or systemic illness. Exclusion criteria included MRI contraindications, BMI > 30 kg/m², high levels of physical activity (> 2 h/week), prior experience with the dynamic balancing task (DBT), and past or current participation in performance-oriented endurance, balance-, or coordination-intensive sports.
Recruitment	Participants were recruited via word of mouth, flyers and posters on campus, announcements on the research group's website, and presentations in university courses. All participants provided written informed consent. Potential self-selection bias may exist (e.g., individuals interested in motor learning or with higher availability for repeated MRI sessions), but the within-subject design minimizes the likelihood of systematic bias in the main findings.
Ethics oversight	The study protocol was reviewed and approved by the Ethics Committee of Otto von Guericke University Magdeburg (reference number 106/98). All participants provided written informed consent prior to participation, in accordance with the Declaration of Helsinki.

Note that full information on the approval of the study protocol must also be provided in the manuscript.

Field-specific reporting

Please select the one below that is the best fit for your research. If you are not sure, read the appropriate sections before making your selection.

☒ Life sciences ☐ Behavioural & social sciences ☐ Ecological, evolutionary & environmental sciences

For a reference copy of the document with all sections, see nature.com/documents/nr-reporting-summary-flat.pdf

Life sciences study design

All studies must disclose on these points even when the disclosure is negative.

Sample size	No a priori power calculation was performed. The target sample size (n = 24, within-subject) was determined by feasibility and scanner time constraints, and aligns with earlier reports on DBT-induced white matter plasticity. Taubert et al. JNeurosci 2010; Lehmann et al. JNeurosci 2020). The within-subject longitudinal design with an explicit non-training control phase (MRI1–MRI2) increases power by reducing inter-individual variance, and multivariate modeling (RM-ASCA+) leverages shared variance across imaging metrics, providing sufficient sensitivity (and enhanced specificity) to detect learning-related effects of the expected magnitude.
Data exclusions	A total of 26 participants were originally enrolled. In 2 participants, diffusion MRI data could not be analyzed due to technical issues during image acquisition. Because the statistical framework required complete multimodal datasets for each individual, these participants were excluded from the final analyses. The remaining 24 participants completed the full study protocol and were included in all reported analyses. Additional quality control at the scan level (visual inspection, SNR, motion artifacts) was performed, but no further participants were excluded.
Replication	All MRI acquisitions and preprocessing steps followed standardized and reproducible protocols (FSL, MRtrix3, TractSeg, hMRI toolbox). Visual inspection and automated quality control ensured consistent data quality across participants and sessions. The within-subject design included a no-intervention control phase (MRI1–MRI2), which served to replicate and validate the stability of imaging measures prior to training. (published in Lehmann et al., Neuroscience, 2021; Aye et al., NeuroImage, 2022). Robustness of statistical results was further confirmed using bootstrapping. No independent replication cohort was included.
Randomization	Randomization was not applicable, as the study used a within-subject longitudinal design in which all participants underwent both a no-intervention control phase (MRI1–MRI2) and a training phase (MRI2–MRI3). The order of phases was fixed to allow within-subject comparison of test–retest reliability versus training-induced changes. No random allocation to experimental groups was performed.
Blinding	Participants were aware of their participation in the training intervention; blinding at this level was not possible. MRI acquisition and preprocessing were fully automated and independent of behavioral outcomes. Statistical analyses were conducted using scripted pipelines in R, ensuring reproducibility and minimizing bias. Investigators were not blinded to the study hypotheses during data analysis.

Reporting for specific materials, systems and methods

We require information from authors about some types of materials, experimental systems and methods used in many studies. Here, indicate whether each material, system or method listed is relevant to your study. If you are not sure if a list item applies to your research, read the appropriate section before selecting a response.

Materials & experimental systems

n/a	Involved in the study
☒	☐ Antibodies
☒	☐ Eukaryotic cell lines
☒	☐ Palaeontology and archaeology
☒	☐ Animals and other organisms
☒	☐ Clinical data
☒	☐ Dual use research of concern
☒	☐ Plants

Methods

n/a	Involved in the study
☒	☐ ChIP-seq
☒	☐ Flow cytometry
☐	☒ MRI-based neuroimaging

Plants

Seed stocks

Report on the source of all seed stocks or other plant material used. If applicable, state the seed stock centre and catalogue number. If plant specimens were collected from the field, describe the collection location, date and sampling procedures.

Novel plant genotypes

Describe the methods by which all novel plant genotypes were produced. This includes those generated by transgenic approaches, gene editing, chemical/radiation-based mutagenesis and hybridization. For transgenic lines, describe the transformation method, the number of independent lines analyzed and the generation upon which experiments were performed. For gene-edited lines, describe the editor used, the endogenous sequence targeted for editing, the targeting guide RNA sequence (if applicable) and how the editor was applied.

Authentication

Describe any authentication procedures for each seed stock used or novel genotype generated. Describe any experiments used to assess the effect of a mutation and, where applicable, how potential secondary effects (e.g. second site T-DNA insertions, mosiacism, off-target gene editing) were examined.

Magnetic resonance imaging

Experimental design

Design type

Within-subject longitudinal design with three MRI sessions at baseline, after a four-week control period, and after a four-week motor learning intervention.

Design specifications

Each participant served as their own control, allowing both test–retest reliability assessment and detection of training-induced white matter changes. Behavioral outcomes (balance time) were modeled with a general power function; imaging data were analyzed with linear mixed models and RM-ASCA+ to capture multivariate longitudinal effects.

Behavioral performance measures

The primary behavioral outcome was balance time, defined as the cumulative duration (in seconds) per 30-s trial during which the platform remained within ±3° of horizontal. Performance feedback was limited to verbal reporting of balance time after each trial, without instruction on movement strategies. Individual learning trajectories across the eight training sessions were modeled using a general power function fitted to within-session averages. These learning rates were subsequently correlated with imaging-derived latent scores of white matter plasticity.

Acquisition

Imaging type(s)

Diffusion-weighted imaging (multishell, 228 directions), multiparameter mapping (MT, PD, R1, R2*), and T1-weighted MPRAGE for anatomical reference. Only diffusion and MPM data were used in the analyses; the MPRAGE served exclusively for intermodal registration.

Field strength

3 T

Sequence & imaging parameters

(non-diffusion only)
Multiparameter mapping employed three multi-echo 3D FLASH acquisitions (T1w: TR = 23 ms, α = 25°, 8 echoes; PDw: TR = 23 ms, α = 5°, 8 echoes; MTw: TR = 37 ms, α = 7°, 6 echoes) at 0.8 mm isotropic resolution (FOV = 230 × 230 mm², 224 sagittal slices). B1+/B1– calibration scans were acquired before each weighted scan. A T1-weighted MPRAGE (~1 mm isotropic) was additionally acquired for anatomical reference and used only for intermodal registration.

Area of acquisition

whole-brain

Diffusion MRI ☒ Used ☐ Not used

Parameters	A monopolar single-shot spin-echo EPI sequence was used with the following parameters: TE = 74 ms, TR = 4970 ms, flip angle = 90°, voxel size = 1.6 × 1.6 × 1.6 mm³, FOV = 208 × 208 mm², matrix = 130 × 130, GRAPPA = 2, multiband factor = 2. The diffusion scheme comprised 228 directions distributed across three shells (b = 1000, 2000, 3000 s/mm²; 38 + 76 + 114 directions), plus 14 interleaved b = 0 volumes. To correct for susceptibility-induced distortions, 9 additional b = 0 volumes were acquired with reversed phase-encoding (posterior-to-anterior). Total acquisition time ≈ 22.5 min.

Preprocessing

Preprocessing software	Diffusion-weighted images were preprocessed in FSL (v6.0; topup, eddy) with gradient rotation according to Leemans & Jones (2009). Tractography and tract segmentation were performed with MRtrix3 and TractSeg v2.8. Microstructural model fitting used the NODDI Matlab Toolbox v1.0.1. Multiparameter maps (MT, PD, R1, R2*) were processed in MATLAB R2022b using the hMRI toolbox v0.2.0 within SPM12, with R2* estimated using the ESTATICS model. Coregistration was performed with FSL FLIRT and boundary-based intermodal registration (bbregister, FreeSurfer).
Normalization	Diffusion data (FA) from the pre-learning session (MRI2) were rigidly registered to the FA template in MNI space using FSL FLIRT, following the TractSeg recommended pipeline. Tract orientation maps were derived from diffusion MRI2 using TractSeg. Microstructural maps from all sessions were projected into MRI2 space to ensure anatomically consistent sampling across timepoints.
Normalization template	Fractional anisotropy (FA) template in MNI space distributed with TractSeg (https://github.com/jelleman8/TractSeg/blob/master/resources/MNI_FA_template.nii.gz)
Noise and artifact removal	For diffusion MRI, all datasets were visually inspected, and images were corrected for susceptibility-induced distortions using topup and for eddy current–induced distortions and motion using eddy in FSL. Gradient vectors were rotated during motion correction. For multiparameter mapping, all quantitative maps were visually inspected, and scans with motion artifacts or intensity outliers were excluded based on standardized SNR criteria. PD maps were corrected for instrumental bias using the B1– field map. R2* maps were estimated with the ESTATICS model, which reduces sensitivity to noise across multi-echo acquisitions.
Volume censoring	Not applicable. No volume censoring was performed, as the study did not include fMRI data. Motion correction in diffusion MRI was handled by FSL eddy.

Statistical modeling & inference

Model type and settings	Analyses were performed at the tract-segment level. For each imaging modality on each tract segment, linear mixed models (time as fixed effect, subject ID as random intercept) were fitted. The resulting fixed-effect estimates across microstructural metrics were assembled into effect matrices and entered into Repeated-Measures ASCA+ (RM-ASCA+), which applies principal component analysis (PCA) to extract latent temporal patterns (primarily PC1). Robustness of the results was assessed with nonparametric bootstrapping (1,000 iterations) and confidence intervals. For behavior–imaging associations, rank correlations of PC1 scores with learning rates were computed and combined across time (MRI1-MRI3, MRI2-MRI3) intervals using the Olkin–Pratt method. For cross-tissue plasticity correlation analyses (white matter PC1 scores with gray matter microstructural changes), robust linear regression with MM estimation was applied, and model significance was evaluated using bias-corrected and accelerated bootstrapping (1,000 samples).
Effect(s) tested	The main effect of interest was longitudinal change in a latent variable derived from RM-ASCA+ (the first principal component, PC1) representing multivariate white matter microstructure. Effects were tested at the segment level for each tract of interest, with a focus on training-related changes (MRI3) compared against stability during the control phase (MRI1 & MRI2). Statistical significance in RM-ASCA+ was inferred from non-overlapping 95% bootstrap confidence intervals for factor scores and loadings. Secondary analyses tested (i) correlations between RM-ASCA+ derived PC1 scores and individual learning rates on the dynamic balancing task, (ii) the dimensionality of latent segment-wise white matter changes (i.e., whether they covary as part of a shared structural network), and (iii) correlations between latent white matter plasticity and previously reported neocortical microstructural plasticity in the same sample.

Specify type of analysis: ☐ Whole brain ☒ ROI-based ☐ Both

Anatomical location(s)	Thirty-five white matter tracts were analyzed, selected based on neurobiological plausibility and prior research on dynamic balance training. These included:<br>Cerebellar pathways: middle cerebellar peduncle, bilateral inferior cerebellar peduncles, bilateral superior cerebellar peduncles<br>Brainstem/cortical motor pathways: bilateral corticospinal tracts, bilateral frontopontine tracts<br>Thalamocortical pathways: bilateral anterior thalamic radiations, superior thalamic radiations, and thalamopremotor tracts<br>Striatal pathways: striato-fronto-orbital, striatopremotor<br>Association tracts: bilateral superior longitudinal fasciculi I–III<br>Corpus callosum segments: rostrum, genu, rostral body, anterior midbody
Statistic type for inference<br>(See Eklund et al. 2016)	Not applicable to voxel- or cluster-wise inference. Statistical tests were performed at the tract-segment level. Inference in RM-ASCA+ was based on bootstrap-derived 95% confidence intervals for latent factor scores and loadings (significance = non-overlapping CIs).
Correction	Conventional voxel- or cluster-wise multiple-comparison correction was not applicable, as analyses were restricted to tract-segment level models within a prespecified set of white matter tracts and inference was based on latent variables derived from RM-ASCA+. In RM-ASCA+, statistical inference relies on bootstrap-derived confidence intervals for factor scores and loadings rather than p-values, such that classical multiple-comparison-correction cannot be applied. To minimize false

positives and ensure behavioral relevance, we therefore adopted a deliberately conservative multi-step strategy: significant latent changes (PC1 from RM-ASCA+) were required to show non-overlapping 95% CIs between the post-learning measurement (MRI3) and both pre-learning measurements (MRI1 and MRI2); segments meeting this criterion were further tested for correlations with individual learning rates in the dynamic balancing task, and only such segments significantly correlating with DBT learning were retained for interpretation. The risk of false positives was quantified via Monte Carlo simulation under a conservative null model.
A strong case for the validity of the results is further supported by the fact that the observed white matter plasticity was strongly interrelated across distant tracts (suggesting network-level reorganization), and converged with independently observed neocortical microstructural alterations in motor-relevant brain areas.

Models & analysis

n/a	Involved in the study
☒	☐ Functional and/or effective connectivity
☒	☐ Graph analysis
☐	☒ Multivariate modeling or predictive analysis

Multivariate modeling and predictive analysis

Multivariate analyses were performed using RM-ASCA+, which combines linear mixed models with principal component analysis to capture latent patterns of longitudinal change in tract-segment microstructure (see details above, “Statistical modeling & inference”) & "Correction"). Out-of-sample analysis (LOOCV) was performed for the regression analysis predicting neocortical neurite orientation dispersion from white matter microstructure.
